# Genetic Variation of the Human Urinary Tract Innate Immune Response and Asymptomatic Bacteriuria in Women

**DOI:** 10.1371/journal.pone.0008300

**Published:** 2009-12-15

**Authors:** Thomas R. Hawn, Delia Scholes, Hongwei Wang, Sue S. Li, Ann E. Stapleton, Marta Janer, Alan Aderem, Walter E. Stamm, Lue Ping Zhao, Thomas M. Hooton

**Affiliations:** 1 Department of Medicine, University of Washington, Seattle, Washington, United States of America; 2 Group Health Center for Health Studies, Group Health Cooperative, and Department of Epidemiology, University of Washington, Seattle, Washington, United States of America; 3 Fred Hutchinson Cancer Research Center, Seattle, Washington, United States of America; 4 Institute for Systems Biology, Seattle, Washington, United States of America; 5 Department of Medicine, University of Miami, Miami, Florida, United States of America; New York University, United States of America

## Abstract

**Background:**

Although several studies suggest that genetic factors are associated with human UTI susceptibility, the role of DNA variation in regulating early *in vivo* urine inflammatory responses has not been fully examined. We examined whether candidate gene polymorphisms were associated with altered urine inflammatory profiles in asymptomatic women with or without bacteriuria.

**Methodology:**

We conducted a cross-sectional analysis of asymptomatic bacteriuria (ASB) in 1,261 asymptomatic women ages 18-49 years originally enrolled as participants in a population-based case-control study of recurrent UTI and pyelonephritis. We genotyped polymorphisms in CXCR1, CXCR2, TLR1, TLR2, TLR4, TLR5, and TIRAP in women with and without ASB. We collected urine samples and measured levels of uropathogenic bacteria, neutrophils, and chemokines.

**Principal Findings:**

Polymorphism TLR2_G2258A, a variant associated with decreased lipopeptide-induced signaling, was associated with increased ASB risk (odds ratio 3.44, 95%CI; 1.65–7.17). Three CXCR1 polymorphisms were associated with ASB caused by gram-positive organisms. ASB was associated with urinary CXCL-8 levels, but not CXCL-5, CXCL-6, or sICAM-1 (P≤0.0001). Urinary levels of CXCL-8 and CXCL-6, but not ICAM-1, were associated with higher neutrophil levels (P≤0.0001). In addition, polymorphism CXCR1_G827C was associated with increased CXCL-8 levels in women with ASB (P = 0.004).

**Conclusions:**

TLR2 and CXCR1 polymorphisms were associated with ASB and a CXCR1 variant was associated with urine CXCL-8 levels. These results suggest that genetic factors are associated with early *in vivo* human bladder immune responses prior to the development of symptomatic UTIs.

## Introduction

Over 100 years ago, scientists observed that bacteriuria could occur in the absence of urinary tract inflammation [1]. This simple fact suggests that although bacteria are capable of inducing bladder inflammation, their presence alone may not necessarily be sufficient to produce an inflammatory response. Thus, bladder inflammation may be regulated by variation in the host response. Although urine is usually sterile, bacteriuria at ≥10^5^ colony-forming units (cfu)/mL is found in approximately 5–10% of healthy, asymptomatic pre-menopausal women [2], [3], [4]. Quantification of bacteriuria was established over 50 years ago as a means to differentiate true bladder bacteriuria from contamination with perineal flora [1], [5], [6]. Several studies demonstrated that asymptomatic bacteriuria (ASB) at ≥10^5^ cfu/ml is associated with an increased risk of developing a symptomatic urinary tract infection (UTI) [2], [3], [4]. Despite its prevalence, the pathogenesis of ASB and risk factors for progression to acute UTI are poorly understood [2], [4], [7], [8]. Similar to acute UTI, ASB has been associated with behavioral risk factors such as sexual activity and contraceptive choice (diaphragm and spermicide use) [4], [9]. However, the majority of women have no obvious functional or anatomic risk factors predisposing them to ASB, which suggests that genetic factors may modulate the risk of acquiring ASB.

After colonization of the urethra and ascent to the bladder, bacteria bind to glycosphingolipid and glycoprotein receptors on urinary tract epithelium and are detected by the innate immune system through pattern recognition receptors such as Toll-like receptors (TLRs) [10], [11]. *E. coli*, which causes 70–90% of all uncomplicated UTIs, is recognized by several TLRs, including TLR1,2,4,5,6 (in humans and mice) and 11 (in mice) [12], [13], [14]. TLRs are located on the cell surface or within organelles such as phagosomes, where they detect microbial ligands such as flagellin (TLR5), lipopolysaccharide (LPS) (TLR4), and bacterial lipopeptides (TLR1/2/6) [10]. Although previous studies in mice indicate that TLRs regulate susceptibility to cystitis and pyelonephritis, TLR modulation of human *in vivo* bladder immune responses is poorly understood [11], [15], [16], [17].

After activation, TLRs mediate signaling pathways that include activation of NF-κB and secretion of chemokines such as CXCL-8 (also called IL-8) [10], [18]. Several clinical studies have demonstrated CXCL-8 in the urine of individuals with acute, symptomatic UTI [19], [20], [21], [22], [23], [24], [25], [26], [27], [28]. Neutrophils migrate from blood vessels to the bladder or kidneys in response to CXCL-8, which binds to the CXCR1 and CXCR2 receptors (also called IL-8RA and IL8RB) [29], [30]. Mice with defects in the IL-8 receptor homologue (mIL-8Rh, homologous to CXCR1 and CXCR2 in humans) are more susceptible to UTIs [29]. After reaching the bladder lumen, neutrophils bind, phagocytose, and eliminate the bacteria. In addition to CXCL-8, several other chemokines are secreted by epithelial cells and induce neutrophil migration including CXCL-5 (also called ENA-78 (Epithelial Cell-Derived Neutrophil-Activating Protein-78)) and CXCL-6 (also called GCP2 (Granulocyte Chemotactic Protein 2)), which also bind to CXCR1/2 [18]. Additional chemotactic molecules operate in blood vessels including sICAM-1 (soluble intercellular adhesion molecule 1), which binds to the CD11b/CD18 (Mac-1) integrin receptor and induces transendothelial migration of neutrophils [31]. Together, these data suggest that several molecules and inflammatory pathways are important in neutrophil migration into the bladder and thus UTI pathogenesis.

A series of studies over several decades indicates that host genetic factors influence susceptibility to human infections [32], [33], [34]. More recent studies suggest an influence of genetics on susceptibility to UTIs [35], [36], [37]. In one family study, 15% of relatives of pyelonephritis-prone children had a UTI history compared to 3% of relatives of controls [36]. We previously found that adult women with rUTI or pyelonephritis were more likely to have a mother with a UTI history in comparison to controls [38], [39]. Susceptibility to rUTI and/or pyelonephritis in humans is associated with polymorphisms in the genes for non-secretor blood group antigens, P1 phenotype, CXCR1, TLR1, TLR2, TLR4, and TLR5 [37], [40], [41], [42], [43], [44], [45], [46], [47], [48], [49]. In addition, reduced expression levels of CXCR1 and CXCR2 on neutrophils was associated with pyelonephritis and recurrent cystitis, respectively [29], [37], [50], [51]. We recently found associations of variants of TLRs 1, 4, and 5 with susceptibility to cystitis and/or pyelonephritis in a case-control study of adult women [49]. Studies of genetic variation and ASB have been more limited, with one previous study demonstrating an association of SNP TLR2_G2258A with ASB in children [47]. Reduced expression levels of TLR4 were associated with an increased risk of ASB [50]. To our knowledge, no studies have examined whether polymorphisms are associated with bacteriuria or altered urinary chemokine or neutrophil levels in asymptomatic adults.

In this study, we examined whether CXCR1, CXCR2, and TLR pathway polymorphisms are associated with ASB, urinary chemokines, and neutrophils in a cross-sectional study of adult women ages 18–49 years with and without a history of recurrent cystitis or pyelonephritis. Asymptomatic bacteriuria provides an opportunity to investigate the role of genetic factors in the early phases of the human *in vivo* host immune response to bacteria.

## Materials and Methods

### Study Setting and Participants

The study protocols were approved by the Human Subjects committees at Group Health Cooperative, the University of Washington, and Western Institutional Review Board. The study was conducted at Group Health Cooperative in Seattle, Washington. Written informed consent was obtained from all subjects. We performed a cross-sectional study to examine whether candidate gene polymorphisms are associated with ASB or urine inflammatory markers in 1261 women. The subjects were enrolled as part of a case-control study of rUTI and pyelonephritis that has been previously described [49]. Briefly, we selected potential rUTI and pyelonephritis cases from the health plan's automated databases identified through having received an International Classification of Diseases (ICD-9) diagnosis code. Recurrent cystitis (rUTI) case subjects (n = 431) were identified based on 3 diagnosed UTI episodes within a 12-month time frame or 2 UTIs within 6 months (episodes were separated by at least 30 days). Culture confirmation (≥10^3^ cfu/mL of a urinary pathogen) or UTI guideline-related treatment was required for all UTI episodes in the cluster. Women experiencing previous pyelonephritis (n = 400) were identified through having received a pyelonephritis ICD-9 diagnosis code and, if they received only outpatient treatment, a primary diagnosis of pyelonephritis and an accompanying culture result of ≥10^3^ cfu/mL of a urinary pathogen or accompanying antibiotic therapy appropriate for pyelonephritis. The remainder of the women constituted the potential control subjects (n = 430) with no history of UTIs who were randomly selected and frequency-matched by case age group (age categories were 18–29, 30–39, 40–49 years). For these analyses, the 1261 participants, all asymptomatic at their study clinic visits, were classified into groups of ASB status based on the urine bacterial counts.

### Urine Specimen Collection and Processing

Subjects were asymptomatic at the time of their clinic visits. We collected a clean-catch midstream urine specimen in a sterile BD Vacutainer™ Urine Tube (Becton, Dickinson and Company) containing a lyophilized preservative including boric acid and sodium formate. Specimens were stored at room temperature and transported to the laboratory within 72 hours where they were inoculated onto blood agar, MacConkey, and CNA plates. Plates were incubated at 37°C for 48 hours, and organisms were quantified in colony-forming units (cfu)/ml and further identified using standard methods [52]. All uropathogens present in midstream urine at ≥10^3^ cfu/ml were identified and quantified and *E. coli* were characterized as beta- or non-hemolytic. A second urine sample was collected in a sterile container to measure urinary neutrophil levels with a dipstick assay (Bayer Multistix Reagent Strips) and reported on a scale of negative, trace, small (+1), moderate (+2), or large (+3). The trace category was excluded from the analysis. The negative category was compared to the 3 other values. In addition, 2 mls of urine from the 2^nd^ collection were immediately frozen and stored at −70°C for later testing for IL-6, CXCL-8, CXCL-5, CXCL-6, and sICAM-1 levels. Prior to testing, specimens were centrifuged at 12,000 RPM for 2 minutes, and the supernatants were removed for analysis. The supernatant was subjected to a sandwich ELISA technique (Duoset, R&D Systems, Minneapolis, MN). Values below the lowest detectable limit of the assay were replaced as zero (lowest detectable values for CXCL-8, CXCL-5, CXCL-6, sICAM-1 were 32.5, 16.1, 6.1, and 6.1 respectively).

### Genomic Techniques

Genomic DNA was purified from peripheral blood by QIAamp DNA blood kit (Qiagen). For genotyping in the full cohort, we generated haplotype-tagging SNPs from our sequencing data as well as publicly available data from the Innate Immunity Program in Genomic Applications (IIPGA, http://innateimmunity.net/). For determining haplotype tagging SNPs, we selected polymorphisms in the chromosomal region of each gene which represented the commonly inherited haplotypes (combinations of individual alleles). Selection criteria were based on linkage disequilibrium measurements between the polymorphisms [53]. Genotyping was carried out with a MassARRAY^TM^ technique (Sequenom) as previously described [54], [55].

### Statistical Analyses

Several different exposures and outcomes were assessed in the analysis. For the primary analysis, polymorphisms of candidate genes were classified as exposures and ASB or urine inflammatory markers (chemokines or neutrophils) were treated as the outcomes. To evaluate the association of genetic polymorphisms with ASB, we defined 3 outcome groups: no ASB (<10^3^ CFU/ml of uropathogen), medium ASB (≥10^3^ and <10^5^ CFU/ml of uropathogen) and high ASB (≥10^5^ CFU/ml of uropathogen). We also evaluated ASB as an exposure with urine neutrophils or chemokines as the outcomes. For these analyses, only cultures with uropathogens were examined. Cultures with non-uropathogens (coagulase-negative staphylococci, other gram-negative rods, and mixed gram-positive flora) were not included in the definition of ASB. Furthermore, any culture with greater than or equal to 100,000 CFU/ml of coagulase-negative staphylococci, mixed gram-negative flora, or mixed gram-positive flora was excluded from the analysis to avoid samples that may have been contaminated with skin or perineal flora during collection (n = 139 excluded and n = 1122 analyzed further). Gram-negative uropathogens included *E. coli* non-hemolytic, *E. coli* β-hemolytic, *Proteus* species, *Enterobacter* species, *Klebsiella* species, *Citrobacter* species, and *Morganella morganii*. Gram-positive uropathogens included *Enterococcus* species, *Lactobacillus* species, Group B Streptococcus, *Staphylococcus aureus*, and *S. saprophyticus*. In addition to stratifying by ASB level, we performed subgroup analyses of ASB caused by gram-negative and gram-positive pathogens.

For the genetic analyses, SNP genotypes (the exposure) and ASB status or (the outcomes) were examined with an allelic trend test. In the allelic trend test (also called a log-additive model) common homozygous genotypes (00) were assigned a value of 0, heterozygotes (01) a value of 1, and minor homozygous genotypes (11) a value of 2. Odds ratios and statistical significance values were then assessed using logistic regression models. We analyzed the ASB groups separately (medium ASB vs no ASB or high ASB vs no ASB) and as a combined group (medium + high ASB vs no ASB). Two-sided testing was used for all comparisons to evaluate statistical significance, considering a P-value of ≤0.05 as significant. To verify that our significant findings were not due to population admixture, we also performed Caucasian subgroup analyses. All analyses were performed using the software Hplus or SAS [56]. Hardy-Weinberg equilibrium (HWE) testing was performed for all SNP genotypes using Haploview. All SNP genotypes satisfied Hardy-Weinberg in the Caucasian control group indicating that there were no genotyping errors or major effects of population heterogeneity.

Univariate analyses were performed for categorical variables with a Chi-Square test; Fisher's exact test was used when the number of samples in a group was less than 5. The Mann-Whitney U-test was used to make comparisons of the chemokine levels between ASB Groups. We expressed the chemokine data as 75th or 90th percentile values due to the high number of women with undetectable urinary chemokines. The percent of women with undetectable chemokine levels was: 32.6% for CXCL-8, 39.1% for CXCL-5, 39.5% for CXCL-6, and 5.8% for sICAM-1. Chemokine levels were analyzed by genotype for each polymorphism with an allelic trend test using a general linear model.

While these data were analyzed cross-sectionally, the study sample was originally selected for a study employing a case-control methodology, and thus case-control status could potentially confound the associations of current interest. To address this issue, we adjusted our analyses for case-control status, finding that this design variable did not act as a confounder. We also incorporated an interaction term for case-control status and the exposures of interest, to assess possible heterogeneity in associations.

## Results

### Study Population Characteristics

We used a cross-sectional approach to examine whether candidate gene polymorphisms were associated with bacteriuria, urine chemokines, or urine neutrophils. We also examined whether bacteriuria was associated with chemokine or neutrophil levels. Midstream voided urines were obtained from 1261 women during their asymptomatic enrollment clinic visits and used to define the three groups: no ASB (<10^3^ cfu/ml), medium ASB (≥10^3^ to <10^5^ cfu/ml), or high ASB (≥10^5^ cfu/ml). The ethnic backgrounds of women in these 3 groups were similar with a predominance of a Caucasian background (Caucasian frequency 77.6% in no ASB group, 74.1% in medium ASB group, and 84.8% in high ASB group (Table 1) The history of co-morbid conditions and urinary tract procedures was similar except that diabetes was more common in women with ASB (P≤0.01 for those with medium ASB and P≤0.05 for those with high ASB) and bladder or kidney surgery was more common in those with high ASB (P≤0.01) (Table 1). The predominant organisms were *E. coli* and *Enterococcus* with smaller numbers of various other bacteria (Table 1).

**Table 1:** Demographic and microbiologic characteristics of ASB study groups.

Variable	No ASB a (N = 731)	Medium ASB (N = 286)	High ASB (N = 105)
	N (%)^a^	N (%)^a^	N (%)^a^
Age at study enrollment, years (mean)	36.6	38.6	39.3
Ethnicity^b^			
American Indian/Alaska native	30 (4.1)	10 (3.5)	4 (3.8)
Asian	85 (11.6)	39 (13.6)	8 (7.6)
Black or African American	48 (6.6)	20 (7.0)	6 (5.7)
Native Hawaiian or Pacific Is.	13 (1.8)	9 (3.2)	2 (1.9)
Caucasian	567 (77.6)	212 (74.1)	89 (84.8)
Hispanic/Latino	56 (7.7)	17 (5.9)	9 (8.6)
Other	40 (5.5)	14 (4.9)	4 (3.8)
Health conditions (history of)			
Kidney stones	29 (4.0)	19 (6.6)	6 (5.7)
Kidney failure/insufficiency	4 (0.6)	1 (0.4)	2 (1.9)
Diabetes (not during pregnancy)	12 (1.6)	15 (5.2)^c^	5 (4.8)^d^
Urinary Tract Procedure History			
Bladder/kidney surgery	30 (4.10)	14 (4.90)	14 (13.33)^c^
Cystoscopy	41 (5.61)	17 (5.94)	10 (9.52)
**Microbiology** e			
Gram-Negative Bacteria			
*E. coli*: beta hemolytic		39	7
*E. coli*: non beta hemolytic		65	33
*Enterobacter* species		5	0
*Proteus* species		0	0
*Proteus mirabilis*		11	3
*Klebsiella* species		11	9
*Citrobacter* species		5	4
*Morganella* species		2	0
Other Gram-negative Rods f		1	0
Gram-Positive Bacteria			
*Enterococcus* species		160	53
Group B Streptococcus		53	13
Lactobacillus		19	22
*Staphylococcus saprophyticus*		0	1
*Staphylococcus aureus*		2	1
*Staphylococcus*, coagulase-negative f		1	1
Mixed Gram Positive Flora (MGP)^f^		638	0

a Definitions: CFU/ml of uropathogen: No ASB <10^3^; Medium ASB: ≥10^3^ & <10^5^; High ASB: ≥10^5^.

b Numbers and percentages in ethnicity subcategories can be greater than total number due to selection of more than one category for an individual.

C P≤0.01 or ^d^ P≤0.05, when compared to group with no ASB.

e For microbiology, numbers without percentages listed. The number of samples without growth of a specific pathogen in the “No ASB” group is left blank since it is the remainder of the 1122 individuals who had culture data available, but did not meet Medium or High ASB criteria.

f Not included in uropathogen definition for this study.

### TLR2 and CXCR1 Polymorphisms Are Associated with ASB

We first examined whether polymorphisms in candidate immune response genes were associated with ASB. We examined 5 polymorphisms in CXCR1, as previous studies have described an association of 2 polymorphisms with an increased risk of pyelonephritis in children [37]. We also examined 5 polymorphisms in CXCR2 since it is a receptor for CXCL-8. One polymorphism (CXCR2_T997C) had no variation and was not examined further. Finally, we examined 7 well-characterized TLR-pathway variants since previous studies suggest that these SNPs are associated with altered TLR gene function and susceptibility to different infections. The polymorphisms include TLR1_G1805T (amino acid (AA) change S602I), TLR2_G2258A (AA R753Q), TLR4_A896G (AA D299G), TLR4_C1196T (AA T399I), TLR5_C1174T (AA R392STOP), TIRAP_C539T (AA S180L), and TIRAP_C558T (AA A186A) [57], [58], [59], [60], [61], [62], [63], [64], [65], [66].

Polymorphism TLR2_2258A, a hyporesponsive allele, was associated with high ASB in that such subjects were more likely to have the minor 2258A allele than those without ASB (Table 2, high vs no ASB, Odds Ratio (OR) 3.44 (95% CI: 1.65–7.17, P = 0.001)). To adjust for population heterogeneity, we analyzed data in the Caucasian subgroup and found a similar association of TLR2_G2258A with high ASB (Table S1, high vs no ASB, OR 3.17 (95% CI: 1.52–6.61, P = 0.002)). When we examined ASB caused by gram-negative uropathogens, the results were similar (Table 3, for high vs no ASB, OR 4.82 (95% CI: 2.06–11.27, P = 0.0003. Also Table S2). We next examined possible confounding factors that might account for this association, including case (rUTI or pyelonephritis) and control (no UTI history) status. A logistic regression model with case-control status as a covariate found a similar association of TLR2_G2258A with ASB caused by gram-negative uropathogens (P = 0.0004, data not shown). In addition, we did not find any interaction between SNP TLR2_G2258A with case-control status (P = 0.964 for interaction).

**Table 2:** CXCR1, CXCR2, and TLR polymorphisms and ASB.

Gene	SNP a		Minor Allele Frequency				Medium vs no ASB^b^		High vs no ASB		combine vs no ASB	
		BP	no ASB	Medium	High	Combine	OR, 95% CI	P	OR, 95% CI	P	OR, 95% CI	P
CXCR1	rs3138060	C/G	0.062	0.070	0.041	0.063	1.16 (0.78, 1.71)	0.470	0.65 (0.31, 1.36)	0.253	1.02 (0.71, 1.47)	0.916
	T92G (rs16858811)	T/G	0.027	0.036	0.029	0.034	1.32 (0.77, 2.29)	0.316	1.08 (0.45, 2.57)	0.869	1.26 (0.76, 2.08)	0.374
	G827C (rs2234671)	G/C	0.072	0.083	0.057	0.076	1.17 (0.82, 1.670	0.396	0.78 (0.42, 1.44)	0.426	1.06 (0.76, 1.48)	0.728
	C1003T (rs16858808)	C/T	0.025	0.034	0.024	0.031	1.33 (0.76, 2.34)	0.316	0.95 (0.37, 2.44)	0.915	1.23 (0.73, 2.070	0.438
	ZA11069G	G/A	0.042	0.027	0.033	0.028	0.62 (0.35, 1.11)	0.107	0.79 (0.36, 1.75)	0.561	0.67 (0.41, 1.10)	0.111
CXCR2	ZC9316T	C/T	0.033	0.029	0.027	0.028	0.89 (0.49, 1.61)	0.692	0.81 (0.32, 2.08)	0.664	0.87 (0.51, 1.48)	0.601
	C768T (rs11574750)	C/T	0.041	0.041	0.033	0.039	0.98 (0.60, 1.61)	0.949	0.80 (0.36, 1.78)	0.586	0.94 (0.60, 1.46)	0.768
	T997C^c^	T/C	0.000	0.000	0.000	0.000						
	ZG12229A	G/A	0.393	0.419	0.406	0.416	1.11 (0.91, 1.36)	0.292	1.06 (0.78, 1.43)	0.726	1.10 (0.92, 1.32)	0.311
	ZT13639C	C/T	0.489	0.459	0.505	0.472	0.89 (0.73, 1.08)	0.231	1.07 (0.80, 1.42)	0.670	0.93 (0.78, 1.11)	0.434
TLR Genes	TLR1_G1805T (rs5743618)	G/T	0.424	0.447	0.341	0.419	1.10 (0.89, 1.35)	0.382	**0.70 (0.51, 0.97)**	**0.033**	0.98 (0.81, 1.18)	0.816
	TLR2_G2258A (rs5743708)	G/A	0.016	0.018	0.052	0.027	1.11 (0.53, 2.35)	0.780	**3.44 (1.65, 7.17)**	**0.001**	1.72 (0.95, 3.14)	0.074
	TLR4_A896G (rs4986790)	A/G	0.056	0.052	0.034	0.047	0.92 (0.60, 1.42)	0.706	0.60 (0.27, 1.31)	0.195	0.83 90.56, 1.24)	0.368
	TLR4_C1196T (rs4986791)	C/T	0.054	0.041	0.038	0.040	0.74 (0.46, 1.19)	0.209	0.69 (0.33, 1.46)	0.332	0.73 (0.47, 1.11)	0.139
	TLR5_C1174T(rs5744168)	C/T	0.046	0.047	0.072	0.054	1.03 (0.65, 1.63)	0.891	1.61 (0.90, 2.88)	0.106	1.19 (0.80, 1.76)	0.401
	TIRAP_C539T(rs8177374)	C/T	0.121	0.120	0.159	0.130	0.97 (0.73, 1.33)	0.926	1.37 (0.91, 2.05)	0.129	1.09 (0.84, 1.41)	0.542
	TIRAP_C558T(rs7932766)	C/T	0.201	0.186	0.204	0.191	0.91 (0.71, 1.17)	0.450	1.02 (0.71, 1.46)	0.926	0.94 (0.75, 1.17)	0.566

a For coding region SNPs, the name includes nucleotide numbering based on mRNA with start codon at 1. For non-coding region SNPs, the name is from the IIPGA database (http://innateimmunity.net/IIPGA2/index_html) and designated with a ‘z” prefix. rs numbers from the dbSNP database are included when available. A log-additive model was used for analysis. P values ≤0.05 in bold.

b no ASB: <10^3^ CFU/ml, medium ASB: >10^3^ and <10^5^ CFU/ml; high ASB: >10^5^ CFU/ml.

c Polymorphism CXCR2_T997C had no variation and could not be analyzed further.

**Table 3:** CXCR1, CXCR2, and TLR polymorphisms and ASB caused by gram-negative uropathogens.

Gene	SNP a		Minor Allele Frequency				Medium vs no ASB^b^		High vs no ASB		combine vs no ASB	
		BP	no ASB	Medium	High	Combine	OR, 95% CI	P	OR, 95% CI	P	OR, 95% CI	P
CXCR1	rs3138060	C/G	0.060	0.084	0.054	0.076	1.45 (0.87, 2.41)	0.152	0.91 (0.36, 2.28)	0.836	1.29 (0.82, 2.04)	0.278
	T92G (rs16858811)	T/G	0.028	0.053	0.011	0.041	**1.98 (1.04, 3.76)**	**0.038**	0.38 (0.05, 2.77)	0.340	1.50 (0.81, 2.77)	0.203
	G827C (rs2234671)	G/C	0.072	0.099	0.051	0.084	1.42 (0.89, 2.28)	0.145	0.70 (0.28, 1.74)	0.437	1.19 (0.77, 1.83)	0.426
	C1003T (rs16858808)	C/T	0.025	0.057	0.010	0.043	**2.40 (1.28, 4.51)**	**0.007**	0.41 (0.06, 3.00)	0.379	1.78 (0.97, 3.27)	0.063
	ZA11069G	G/A	0.038	0.023	0.051	0.031	0.58 (0.23, 1.45)	0.246	1.36 (0.54, 3.44)	0.520	0.81 (0.42, 1.59)	0.547
CXCR2	ZC9316T	C/T	0.034	0.010	0.023	0.014	0.28 (0.07, 1.34)	0.074	0.66 (0.16, 2.74)	0.567	0.29 (0.14, 1.08)	0.069
	C768T (rs11574750)	C/T	0.038	0.072	0.010	0.053	**1.96 (1.12, 3.42)**	**0.019**	0.26 (0.04, 1.89)	0.183	1.41 (0.82, 2.43)	0.211
	T997C^c^	T/C	0.000	0.000	0.000	0.000						
	ZG12229A	G/A	0.397	0.445	0.375	0.424	1.22 (0.91, 1.62)	0.172	0.91 (0.60, 1.39)	0.668	1.12 (0.88, 1.42)	0.374
	ZT13639C	C/T	0.489	0.412	0.541	0.451	**0.73 (0.55, 0.97)**	**0.029**	1.23 (0.82, 1.85)	0.313	0.86 (0.68, 1.09)	0.208
TLR Genes	TLR1_G1805T (rs5743618)	G/T	0.426	0.451	0.296	0.403	1.11 (0.83, 1.48)	0.499	**0.67 (0.36, 0.90)**	**0.017**	0.91 (0.71, 1.18)	0.480
	TLR2_G2258A (rs5743708)	G/A	0.016	0.031	0.071	0.043	1.99 (0.86, 4.57)	0.107	**4.82 (2.06, 11.27)**	**0.0003**	**2.81 (1.48, 5.36)**	**0.002**
	TLR4_A896G (rs4986790)	A/G	0.052	0.054	0.073	0.059	1.04 (0.56, 1.93)	0.900	1.45 (0.65, 3.20)	0.365	1.16 (0.70, 1.92)	0.567
	TLR4_C1196T (rs4986791)	C/T	0.050	0.035	0.061	0.043	0.70 (0.33, 1.45)	0.333	1.24 (0.53, 2.89)	0.627	0.86 (0.48, 1.52)	0.693
	TLR5_C1174T(rs5744168)	C/T	0.051	0.031	0.041	0.034	0.59 (0.27, 1.28)	0.179	0.79 (0.28, 2.19)	0.646	0.65 (0.34, 1.22)	0.177
	TIRAP_C539T(rs8177374)	C/T	0.127	0.097	0.133	0.107	0.73 (0.46, 1.16)	0.185	1.05 (0.58, 1.91)	0.875	0.83 (0.57, 1.20)	0.316
	TIRAP_C558T(rs7932766)	C/T	0.195	0.186	0.271	0.211	0.94 (0.66, 1.34)	0.737	1.53 (0.96, 2.44)	0.072	1.10 (0.83, 1.48)	0.506

a For coding region SNPs, the name includes nucleotide numbering based on mRNA with start codon at 1. For non-coding region SNPs, the name is from the IIPGA database (http://innateimmunity.net/IIPGA2/index_html) and designated with a ‘z” prefix. rs numbers from the dbSNP database are included when available. A log-additive model was used for analysis. P values ≤0.05 in bold.

b no ASB: <10^3^ CFU/ml, medium ASB: >10^3^ and <10^5^ CFU/ml; high ASB: >10^5^ CFU/ml.

c Polymorphism CXCR2_T997C had no variation and could not be analyzed further.

We also found an association of polymorphisms TLR1_G1805T with ASB in an unadjusted analysis (Table 2). However, in the Caucasian subgroup, the TLR1 association was not present (Table S1). We recently found that SNPs TLR5_C1174T and TLR4_A896G were associated with different UTI outcomes [49]. There was no association of these TLR pathway polymorphisms with ASB status from all uropathogens (Table 2). Two TLR4 SNPs were associated with ASB caused by gram-positive organisms (Table 4). The biologic significance of this was not clear since TLR4 recognizes LPS, which is not present on most gram-positive organisms.

**Table 4:** CXCR1, CXCR2, and TLR polymorphisms and ASB caused by gram-positive uropathogens.

Gene	SNP a		Minor Allele Frequency				Medium vs no ASB^b^		High vs no ASB		combine vs no ASB	
		BP	no ASB	Medium	High	Combine	OR, 95% CI	P	OR, 95% CI	P	OR, 95% CI	P
CXCR1	rs3138060	C/G	0.064	0.057	0.056	0.057	0.90 (0.57, 1.42)	0.638	0.87 (0.41, 1.82)	0.701	0.89 (0.59, 1.34)	0.569
	T92G (rs16858811)	T/G	0.025	0.035	0.059	0.042	1.42 (0.78, 2.59)	0.251	**2.44 (1.16, 5.12)**	**0.018**	**1.69 (1.01, 2.82)**	**0.046**
	G827C (rs2234671)	G/C	0.074	0.074	0.067	0.072	1.01 (0.67, 1.51)	0.978	0.89 (0.49, 1.74)	0.741	0.98 (0.68, 1.41)	0.899
	C1003T (rs16858808)	C/T	0.024	0.030	0.053	0.036	1.26 (0.67, 2.38)	0.473	**2.28 (1.05, 4.96)**	**0.038**	1.52 (0.89, 2.60)	0.126
	ZA11069G	G/A	0.042	0.026	0.013	0.022	0.59 (0.31, 1.12)	0.108	0.30 (0.07, 1.24)	0.096	**0.51 (0.28, 0.94)**	**0.030**
CXCR2	ZC9316T	C/T	0.030	0.036	0.030	0.034	1.19 (0.65, 2.19)	0.578	0.99 (0.35, 2.80)	0.989	1.14 (0.65, 1.98)	0.646
	C768T (rs11574750)	C/T	0.039	0.035	0.072	0.045	0.89 (0.50, 1.58)	0.688	1.93 (0.99, 3.74)	0.052	1.15 (0.72, 1.84)	0.552
	T997C^c^	T/C	0.000	0.000	0.000	0.000						
	ZG12229A	G/A	0.395	0.418	0.424	0.419	1.10 (0.88, 1.37)	0.396	1.13 (0.80, 1.59)	0.494	1.11 (0.91, 1.35)	0.309
	ZT13639C	C/T	0.488	0.472	0.461	0.469	0.94 (0.76, 1.16)	0.560	0.90 (0.64, 1.25)	0.519	0.93 (0.77, 1.12)	0.435
TLR Genes	TLR1_G1805T (rs5743618)	G/T	0.419	0.443	0.400	0.432	1.11 (0.88, 1.38)	0.386	0.93 (0.64, 1.34)	0.675	1.06 (0.86, 1.29)	0.591
	TLR2_G2258A (rs5743708)	G/A	0.021	0.012	0.026	0.015	0.54 (0.21, 1.39)	0.202	1.25 (0.44, 3.56)	0.678	0.72 (0.34, 1.51)	0.390
	TLR4_A896G (rs4986790)	A/G	0.058	0.050	0.000	0.037	0.84 (0.52, 1.36)	0.482			**0.61 (0.38, 0.99)**	**0.046**
	TLR4_C1196T (rs4986791)	C/T	0.054	0.042	0.013	0.034	0.76 (0.45, 1.27)	0.289	**0.23 (0.06, 0.95)**	**0.042**	0.62 (0.38, 1.01)	0.055
	TLR5_C1174T(rs5744168)	C/T	0.044	0.056	0.080	0.062	1.27 (0.79, 2.05)	0.316	1.88 (1.00, 3.55)	0.055	1.43 (0.95, 2.15)	0.089
	TIRAP_C539T(rs8177374)	C/T	0.122	0.121	0.160	0.131	0.99 (0.72, 1.37)	0.954	1.37 (0.87, 2.18)	0.179	1.09 (0.82, 1.44)	0.569
	TIRAP_C558T(rs7932766)	C/T	0.204	0.178	0.180	0.179	0.85 90.64, 1.12)	0.236	0.86 (0.56, 1.32)	0.481	0.85 (0.66, 1.08)	0.189

a For coding region SNPs, the name includes nucleotide numbering based on mRNA with start codon at 1. For non-coding region SNPs, the name is from the IIPGA database (http://innateimmunity.net/IIPGA2/index_html) and designated with a ‘z” prefix. rs numbers from the dbSNP database are included when available. A log-additive model was used for analysis. P values ≤0.05 in bold.

b no ASB: <10^3^ CFU/ml, medium ASB: >10^3^ and <10^5^ CFU/ml; high ASB: >10^5^ CFU/ml.

c Polymorphisms CXCR2_T997C and TLR4_A896G (in the High ASB group) had no variation and could not be analyzed further.

We did not find any associations of CXCR1 or CXCR2 polymorphisms with ASB in the entire group or in the Caucasian subgroup (Tables 2 and S1). However, several CXCR1 polymorphisms were associated with ASB caused by gram-positive pathogens (Table 4, SNP T92G, for high ASB, OR 2.44 (95% CI: 1.16–5.12, P = 0.018; SNP C1003T, for high ASB, OR 2.28 (1.05–4.96), P = 0.038); and SNP ZA11069G for all ASB, OR 0.51 (0.28–0.94), P = 0.03)). Two of these associations remained statistically significant when analyzed in the Caucasian only subgroup (Table S3, SNP T92G, for high ASB, OR 2.34 (95% CI: 1.07–5.14, P = 0.035; and SNP ZA11069G for all ASB, OR 0.51 (0.28–0.96), P = 0.036)). One SNP showed a similar magnitude of effect that was not statistically significant (SNP C1003T, for high ASB, OR 2.10 (0.92–4.83), P = 0.080). There were several associations of CXCR1 polymorphisms with ASB caused by gram-negative pathogens (Table 3). However, the biologic significance of these latter associations was less clear since they were only present at medium, but not high, levels of ASB. We also examined whether CXCR1 or CXCR2 polymorphisms were associated with a history of recurrent cystitis (rUTI) or pyelonephritis We did not find any significant associations in the entire group or the Caucasian only subgroup (Tables S4 and S5).

Together, these results provide evidence that polymorphism TLR2_G2258A is associated with ASB (all ASB as well as ASB caused by gram-negative uropathogens) and CXCR1 is associated with ASB caused by gram-positive uropathogens.

### ASB Is Associated with Urine CXCL-8, but Not Other Chemokines

To examine whether ASB is associated with chemokine levels, we measured urine levels of CXCL-8, CXCL-5, CXCL-6, and sICAM-1. We also examined IL-6, but only a small number of women had detectable IL-6 levels (9 of 561during initial testing), so no further measurement or analysis was performed. CXCL-8 was associated with increasing levels of ASB (Table 5, P<0.001 when comparing no ASB (90^th^ percentile value 55.7 pg/ml) vs. medium ASB (102.6 pg/ml) vs. high ASB (249.3 pg/ml). This association was present for ASB caused by both gram-negative as well as gram-positive pathogens. We also examined CXCL-5, CXCL-6, and sICAM-1 and found no difference in these outcomes by womens' ASB status. sICAM-1, in contrast to the other chemokines, had high basal levels in women without ASB. Together, these results suggest a selective association of ASB with CXCL-8, but not CXCL-5, CXCL-6, or ICAM-1.

**Table 5:** ASB and urine chemokine levels.

Chemokine	ASB Type	75th percentile value			90th percentile value			P
		no ASB (n = 731)	Medium ASB (n = 286)	High ASB (n = 105)	no ASB	Medium ASB	High ASB	ASB^a^
CXCL-8 (pg/ml)	All	32.5	32.5	51.2	55.7	102.6	249.3	**<0.001**
	Gram−	32.5	32.5	101.3	60.8	120.7	395.0	**0.004**
	Gram+	32.5	32.5	32.5	71.3	99.6	126.6	**0.0002**
CXCL-5 (pg/ml)	All	18.4	21.6	18.6	34.5	40.3	56.1	0.599
	Gram−	18.1	22.2	29.5	36.9	33.4	62.0	0.707
	Gram+	19.1	22.2	16.1	35.4	44.5	19.4	0.436
CXCL-6 (pg/ml)	All	24.8	20.2	25.9	56.3	44.6	67.1	0.758
	Gram−	23.4	20.2	46.1	51.9	43.6	101.4	0.156
	Gram+	25.2	20.9	18.8	60.9	41.8	40.2	0.284
sICAM-1 (pg/ml)	All	1132.9	1305.8	1278.7	1597.3	1808.5	1972.7	0.100
	Gram−	1182.7	1242.5	1277.1	1643.1	1609.1	2015.4	0.630
	Gram+	1130.0	1305.8	1372.9	1602.9	1808.5	1972.7	0.067

a P value for comparison of 3 ASB clinical groups (no ASB vs medium vs high). P values or comparisons less than 0.05 are in bold.

### ASB, CXCL-6, and CXCL-8 Are Associated with Urine Neutrophil Levels

We next examined whether ASB is associated with neutrophil levels. ASB was associated with urine neutrophil levels with statistically significant results when comparing positive vs. negative levels of urine neutrophils (Table 6, OR 1.66 (95% CI: 1.14–2.41), P = 0.008). We also examined whether chemokines were associated with different neutrophil levels. CXCL-6 and CXCL-8 were associated with increasing levels of urine neutrophils (Table 6, P = 0.0068 & P<0.0001, respectively). The magnitude of the effect was most striking for CXCL-8 with 90^th^ percentile values ranging from 32.5 pg/ml for those without urine neutrophils to 1578.3 pg/ml for those with high levels. CXCL-5 and sICAM-1 were not associated with urinary neutrophil levels. Together, these results demonstrate that CXCL-6 and CXCL-8 levels, but not CXCL-5 or sICAM-1, are associated with urine neutrophil levels.

**Table 6:** Urine neutrophil association with ASB and chemokine levels.

	Urine Neutrophils				
**ASB Level**	**Negative**	**+**	**++**	**+++**	**Positive** b
<1000 (no ASB)	495	47	23	4	74
> = 1000	242	35	20	5	60
OR (95% CI)^a^	1.0	1.52 (0.96–2.42)	1.78 (0.96–3.30)	2.56 (0.68–9.60)	1.66 (1.14, 2.41)
P value		0.075	0.068	0.165	0.008
**Chemokine**	Median/75%/90%				P^c^
CXCL-8	28.6/32.5/32.5	33.6/89.7/171.5	73.3/284.7/751.8	568.0/912.4/1578.3	<0.0001
CXCL-5	16.1/18.0/36.1	16.1/24.6/47.0	16.1/20.3/42.2	16.1/80.0/188.7	0.0809
CXCL-6	16.1/20.2/43.6	16.1/39.2/66.4	16.1/36.0/254.3	48.9/153.3/289.5	0.0068
ICAM-1	453.8/1114.3/1602.9	682.9/1323.5/1604.8	430.9/1274.4/2161.9	979.7/1211.0/1418.9	0.1676

a Comparing Urine PMNs in those with and without ASB with “negative” PMN group as reference.

b Positive = combination of +, ++, and +++ neutrophil counts.

c Comparing chemokine values at different urine neutrophil levels.

### CXCR1 Polymorphism Is Associated with Urine CXCL-8 Level

**Figure 1:**
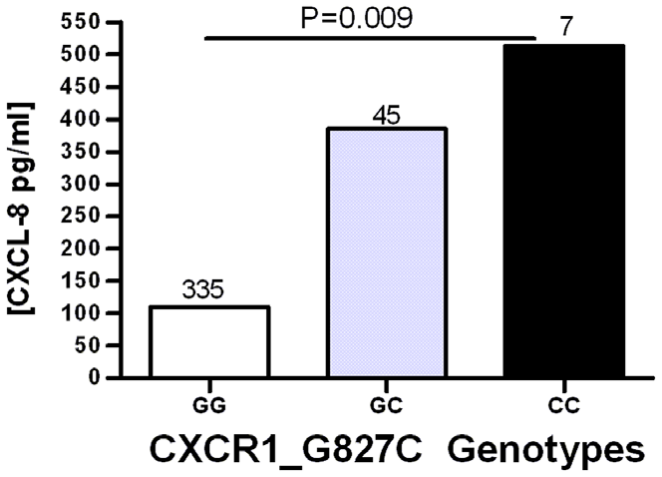
Association of a CXCR1 polymorphism with urine CXCL-8 levels in women with ASB. Urine CXCL-8 levels in women with ASB were stratified by CXCR1_G827C genotypes and analyzed by a log-additive model. The 90^th^ percentile median values are plotted with sample size for each genotype indicated.

Finally, we examined whether DNA polymorphisms were associated with urine chemokine levels in women with ASB. One CXCR1 polymorphism (G827C) was associated with CXCL-8 concentration in women with ASB from all uropathogens (Table 7, P = 0.004). Genotype GG was associated with lower CXCL-8 (110.6 pg/ml 90^th^ percentile) in comparison to genotypes GC or CC (385.6 pg/ml & 513.4 pg/ml, respectively) (Figure 1). We also examined the association of CXCR1_G827C in women with gram-negative and gram-positive ASB and found similar differences (Figure 1). CXCR2_ZT13639C, had a borderline association with CXCL-8 levels (P = 0.05). No other associations were found. Together, these results suggest a selective association of candidate gene polymorphisms with chemokine levels, with the most robust association of a CXCR1 polymorphism with CXCL-8 concentrations in those with ASB.

**Table 7:** CXCR1, CXCR2, and TLR polymorphisms and chemokine levels in women with ASB^a^.

Gene & SNP	Alleles	CXCL-8 [pg/ml]		CXCL-5 [pg/ml]		CXCL-6 [pg/ml]		sICAM-1 [pg/ml]	
	(major/minor)	coefficient	P	coefficient	P	coefficient	P	coefficient	P
CXCR1									
rs3138060	C/G	15.21	0.488	−2.70	0.674	4.83	0.730	42.87	0.669
T92G	T/G	−38.65	0.193	0.48	0.953	−18.72	0.371	−225.38	0.089
G827C	G/C	53.32	**0.009**	−3.05	0.591	−2.49	0.862	139.42	0.125
C1003T	C/T	−39.68	0.208	1.28	0.884	−19.14	0.390	−187.06	0.186
ZA11069G	G/A	−38.99	0.252	−2.54	0.789	−15.21	0.527	203.50	0.181
CXCR2									
ZC9316T	C/T	−52.72	0.176	1.58	0.884	43.79	0.108	−52.36	0.751
C768T	C/T	32.60	0.291	−0.69	0.936	−23.31	0.284	−13.79	0.921
ZG12229A	G/A	7.46	0.513	0.35	0.913	−6.29	0.435	−28.00	0.579
ZT13639C	C/T	−20.96	0.060	0.06	0.985	2.80	0.722	−0.02	1.000
TLR Pathway									
TLR1_G1805T	G/T	8.68	0.468	−1.12	0.737	−3.98	0.634	59.18	0.240
TLR2-G2258A	G/A	45.37	0.212	−8.42	0.406	−19.17	0.455	46.67	0.775
TLR4_A896G	A/G	32.13	0.234	4.96	0.509	0.68	0.972	127.70	0.291
TLR4_C1196T	C/T	24.48	0.420	3.38	0.690	2.78	0.897	−2.28	0.987
TLR5_C1174T	C/T	−2.27	0.932	−2.58	0.727	7.38	0.727	−11.91	0.524
TIRAP_C539T	C/T	10.20	0.533	1.19	0.795	13.72	0.234	−63.19	0.389
TIRAP_C558T	C/T	−7.22	0.624	2.27	0.579	−9.77	0.346	−68.40	0.297

a Women with ASB defined as those with > = 1000 CFU/ml of a uropathogen. Chemokine levels were analyzed by genotype for each polymorphism with a log-additive model.

## Discussion

In this manuscript, we investigated the variation and genetic regulation of inflammation associated with asymptomatic bacteriuria in women in order to gain insight into the early stages of human UTI pathogenesis. Our main findings are that polymorphisms in TLR2 and CXCR1 are associated with ASB and that a CXCR1 polymorphism is associated with urine CXCL-8 levels in women with ASB. In addition, we found that chemokine levels are selectively associated with ASB (CXCL-8, but not CXCL-5, CXCL-6, or ICAM-1) and urinary neutrophil levels (CXCL-6 and CXCL-8, but not CXCL-5 or sICAM-1.

There are several steps in UTI pathogenesis that could be regulated by genetic variation of immune response genes. One of the early steps is the establishment of bacteriuria, which often occurs from ascent of bacteria from the colon, to the periurethral region to the bladder. We found an association of TLR2_G2258A with ASB. Interestingly, this SNP was not associated with a history of rUTI or pyelonephritis in the same cohort [49]. Polymorphism TLR2_2258A encodes an arginine to glutamine amino acid change in the TIR signaling domain, mediates impaired signalling in HEK293 cells transfected with TLR2 in an NF-κB assay, and is associated with decreased cytokine production after *ex vivo* stimulation of whole blood with *Borrelia burgdorferi* lysates [65]. This polymorphism has been associated with protection from Lyme disease as well as increased susceptibility to tuberculosis [65], [67]. A previous study in children found an association of TLR2_G2258A with ASB as well as symptomatic UTIs [47]. Our results confirm these findings with ASB, but not cystitis, susceptibility [49] (39). This apparent contrast in results may be attributable to different study populations (children vs adults from different ethnic backgrounds). Together, these findings suggest that TLR2 signaling may impact susceptibility to human UTIs at an early stage that regulates the establishment of bacteriuria.

In addition, we found a selective association of urinary chemokine levels (CXCL-8, but not CXCL-5, CXCL-6, or ICAM-1) with ASB. Even though CXCL-8, CXCL-5, and CXCL-6 are ligands for CXCR1 and CXCR2, our data suggest differential regulation of these chemokines in response to bacteriuria. With at least 8 chemokines involved in neutrophil chemotaxis, it is often difficult to elucidate specific and/or non-redundant roles of individual chemokines. By focusing on ASB, we attempted to discover which chemokines are involved with neutrophil migration during early steps in UTI pathogenesis. With progression of disease to a symptomatic stage, a different pattern of elevated chemokines likely occurs. Previous studies of urine chemokine levels have mainly focused on IL-6 and CXCL-8 during episodes of symptomatic cystitis [20], [22], [23], [24], [27]. We did not confirm the findings of previous studies of ASB with urine IL-6 levels, but did confirm previous reports of elevated CXCL-8 [19], [21]. Although urine CXCL-5 has been found to be elevated in women with cystitis or urosepsis, it has not been examined in ASB [26], [28]. To our knowledge, CXCL-6, and sICAM-1 have not been measured in previous studies of either symptomatic UTIs or ASB. Further studies of additional chemokines during ASB and other stages of disease should further elucidate the association of specific chemokines with specific steps in UTI pathogenesis.

We also found a selective association of chemokines (CXCL-6, and CXCL-8, but not CXCL-5 or sICAM-1) with urine neutrophil levels. Previous studies demonstrated that urine neutrophil counts are associated with elevated CXCL-8 levels [25]. Depletion of CXCL-8 from urine decreased the chemotactic activity by an average of 54.7%, suggesting that additional chemokines are involved. Our data suggest that CXCL-6 is an additional chemokine involved in chemotaxis of neutrophils to the urine in the presence of bacteriuria. Interestingly, sICAM-1 was not associated with elevated urine neutrophils. sICAM-1 may have a different role in UTI pathogenesis, as it is expressed by endothelial cells rather than bladder epithelium or neutrophils [31]. sICAM-1 binds to MAC1 (CD11b-CD18) on neutrophils and facilitates PMN slowing and subsequent migration from the blood vessel lumen to the interstitial tissue [68]. With a different cell source and bladder location, sICAM-1 likely mediates a different step in PMN migration in comparison to CXCL-5, CXCL-6, and CXCL-8.

We also found an association of several CXCR1 SNPs (T92G, C1003T, and ZA11069G) with ASB caused by gram-positive uropathogens. Furthermore, we found an association of polymorphism G827C in CXCR1 with CXCL-8 levels in women with ASB. This polymorphism (also called variant 2 or 6334:L19592), which encodes a serine to threonine substitution at amino acid 276, has previously been associated with risks of pyelonephritis and vesicoureteral reflux in childhood [37]. The mechanism underlying this association is not clear. One possibility is that CXCR1 variants regulate recruitment of neutrophils to the bladder in response to CXCL-8 produced by epithelial cells or neutrophils. If reduced numbers of neutrophils migrate to the bladder, then one source of CXCL-8 production will be eliminated. Further studies of the effects of CXCR-1_G827C on cyhemokine expression will be needed to clarify this mechanism.

Potential limitations of our study include effects of population admixture and multiple comparisons. To account for possible confounding effects from population heterogeneity, we performed our genetic analyses in the Caucasian subgroup as well as the entire cohort. Despite these attempts to minimize population admixture effects, we cannot exclude this possibility. A second possible limitation is the issue of multiple comparisons. As a matter of hypothesis testing, we prioritized seven well-characterized TLR pathway functional polymorphisms and 10 SNPs in the two major receptors involved in neutrophil chemotaxis. SNPs with well-characterized function do not generally have the same requirement for adjustments for multiple comparisons, due to a well-founded *a priori* hypothesis of their potential association with a cellular function. Even if a strict Bonferroni correction were taken for the association of TLR2_G2258A with ASB, the adjusted P value would remain significant at <0.017 (17 polymorphisms x <0.001 for high vs no ASB comparison). Regardless of which adjustments are chosen for our data, convincing evidence of a genetic effect ultimately requires replication studies as well as detailed analysis of functional effects of each polymorphism. Another limitation is that our cross-sectional study design does not enable us to assess whether the ASB episodes in our population are linked to a future occurrence of cystitis. We recently completed a prospective study of women with recurrent cystitis that included daily urine sampling prior to an episode of cystitis [69]. With such a study design, it may be feasible to analyze whether genetic variation and urine inflammatory profiles associated with ASB are predictive of a subsequent cystitis episode.

Our study also has numerous strengths. First, to our knowledge this is the largest study to examine associations of gene polymorphisms and susceptibility to ASB, and is the only study to date to examine this phenotype in adults. Second, we used a population-based sampling strategy which increases the likelihood that our findings are representative of the population. Finally, this is the first study to examine the association of polymorphisms with urine chemokine and neutrophil levels.

In summary, our results suggest that genetic variation of TLR and chemokine pathways is associated with asymptomatic bacteriuria and urine chemokine levels. Examining genetic regulation of the urinary tract immune response during ASB provides an opportunity to understand early events in pathogenesis prior to the complex cytokine and chemokine inflammatory cascades present during symptomatic cystitis episodes.

## Supporting Information

Table S1 CXCR1, CXCR2, & TLR polymorphisms & ASB (Caucasian only).
(0.22 MB RTF)

Table S2 CXCR1, CXCR2, & TLR polymorphisms & ASB secondary to gram-negative uropathogens for Caucasian only.
(0.18 MB RTF)

Table S3 CXCR1, CXCR2, & TLR polymorphisms & ASB secondary to gram-positive uropathogens for Caucasian only.
(0.17 MB RTF)

Table S4 CXCR1 and CXCR2 polymorphisms & clinical history of rUTI or pyelonephritis.
(0.11 MB RTF)

Table S5 CXCR1 and CXCR2 polymorphisms & clinical history of rUTI or pyelonephritis for Caucasians only.
(0.12 MB RTF)
