# Supplementary material for: Genetic Variation of the Human Urinary Tract Innate Immune Response and Asymptomatic Bacteriuria in Women
Source: PLoS One. 2009 Dec 15;4(12):e8300. doi: 10.1371/journal.pone.0008300 (PMC2788705; doi:10.1371/journal.pone.0008300)
Supplement: Table S1 — CXCR1, CXCR2, & TLR polymorphisms & ASB (Caucasian only). (0.22 MB RTF) [file pone.0008300.s001.rtf]

Table S1: CXCR1, CXCR2, & TLR Polymorphisms & ASB (Caucasian Only)

Gene	SNP a		Minor Allele Frequency	Medium vs no ASBb	High vs no ASB	combine vs no ASB	
		BP	no ASB	Medium	High	Combine	OR, 95% CI	P	OR, 95% CI	P	OR, 95% CI	P	
CXCR1	rs3138060	C/G	0.055	0.063	0.036	0.055	1.15 (0.72, 1.85)	0.560	0.63 (0.27, 1.49)	0.295	1.00 (0.64, 1.55)	0.995	
	T92G (rs16858811)	T/G	0.031	0.031	0.035	0.032	1.03 (0.54, 1.97)	0.929	1.13 (0.47, 2.74)	0.779	1.06 (0.60, 1.88)	0.838	
	G827C (rs2234671)	G/C	0.060	0.057	0.039	0.052	0.95 (0.59, 1.54)	0.834	0.64 (0.29, 1.41)	0.268	0.86 (0.55, 1.32)	0.483	
	C1003T (rs16858808)	C/T	0.029	0.033	0.028	0.032	1.15 (0.61, 2.17)	0.666	0.98 (0.38, 2.53)	0.960	1.10 (0.62, 1.95)	0.748	
													
													
	ZA11069G	G/A	0.052	0.036	0.034	0.035	0.68 (0.38, 1.21)	0.186	0.63 (0.27, 1.49)	0.297	0.66 (0.40, 1.10)	0.115	
													
CXCR2	ZC9316T	C/T	0.037	0.035	0.032	0.034	0.93 (0.49, 1.76)	0.819	0.85 (0.33, 2.20)	0.743	0.91 (0.51, 1.60)	0.734	
	C768T (rs11574750)	C/T	0.046	0.041	0.034	0.039	0.88 (0.50, 1.54)	0.651	0.72 90.31, 1.71)	0.461	0.83 (0.50, 1.37)	0.472	
	T997Cc	T/C	0.000	0.000	0.000	0.000							
													
	ZG12229A	G/A	0.414	0.463	0.395	0.443	1.22 (0.97, 1.53)	0.095	0.92 (0.67, 1.28)	0.638	1.12 (0.92, 1.38)	0.270	
	ZT13639C	C/T	0.483	0.448	0.534	0.473	0.87 (0.69, 1.09)	0.213	1.22 (0.89, 1.68)	0.210	0.96 (0.79, 1.17)	0.695	
TLR Genes	TLR1_G1805T (rs5743618)	G/T	0.305	0.306	0.247	0.289	1.01 (0.78, 1.30)`	0.962	0.75 (0.50, 1.11)	0.148	0.93 (0.73, 1.17)	0.520	
	TLR2_G2258A (rs5743708)	G/A	0.020	0.021	0.062	0.033	1.05 (0.48, 2.28)	0.907	3.17 (1.52, 6.61)	0.002	1.66 (0.90, 3.04)	0.103	
	TLR4_A896G (rs4986790)	A/G	0.062	0.055	0.035	0.049	0.88 (0.54, 1.43)	0.609	0.54 (0.23, 1.26)	0.152	0.78 90.50, 1.21)	0.270	
	TLR4_C1196T (rs4986791)
TLR5_C1174T(rs5744168)	C/T
C/T	0.064
0.045	0.048
0.059	0.034
0.068	0.044
0.062	0.74 (0.44, 1.22)
1.33 (0.81, 2.18)	0.238
0.253	0.51 (0.22, 1.20)
1.55 (0.81, 2.97)	0.124
0.188	0.67 (0.42, 1.06)
1.40 (0.90, 2.16)	0.087
0.134	
	TIRAP_C539T(rs8177374)
TIRAP_C558T(rs7932766)	C/T
C/T	0.144
0.229	0.157
0.223	0.176
0.230	0.163
0.225	1.11 (0.81, 1.51)
0.97 (0.74, 1.26)	0.513
0.793	1.27 (0.83, 1.94)
1.01 (0.69, 1.47)	0.263
0.975	0.16 (0.88, 1.52)
0.98 90.77, 1.24)	0.297
0.845	
													
													
a For coding region SNPs, the name includes nucleotide numbering based on mRNA with start codon at 1.  For non-coding region SNPs, the name is from the IIPGA database (http://innateimmunity.net/IIPGA2/index_html) and designated with a 'z” prefix.  rs numbers from the dbSNP database are included when available.  A log-additive model was used for analysis.  P values ≤ 0.05 in bold.  b no ASB: <103 CFU/ml, medium ASB: >103 and <105 CFU/ml; high ASB: >105 CFU/ml.  cPolymorphism CXCR2_T997C had no variation and could not be analyzed further.
