# Supplementary material for: Genetic Variation of the Human Urinary Tract Innate Immune Response and Asymptomatic Bacteriuria in Women
Source: PLoS One. 2009 Dec 15;4(12):e8300. doi: 10.1371/journal.pone.0008300 (PMC2788705; doi:10.1371/journal.pone.0008300)
Supplement: Table S3 — CXCR1, CXCR2, & TLR polymorphisms & ASB secondary to gram-positive uropathogens for Caucasian only. (0.17 MB RTF) [file pone.0008300.s003.rtf]

Table S3: CXCR1, CXCR2, & TLR Polymorphisms & ASB Secondary to Gram-Positive Uropathogens for Caucasian only
Gene	SNP a		Minor Allele Frequency	Medium vs no ASBb	High vs no ASB	combine vs no ASB	
		BP	no ASB	Medium	High	Combine	OR, 95% CI	P	OR, 95% CI	P	OR, 95% CI	P	
CXCR1	rs3138060	C/G	0.057	0.047	0.058	0.050	0.81 (0.45, 1.46)	0.483	1.03 (0.46, 2.28)	0.951	0.87 (0.53, 1.44)	0.588	
	T92G (rs16858811)	T/G	0.028	0.030	0.064	0.040	1.07 (0.51, 2.24)	0.867	2.34 (1.07, 5.14)	0.035	1.43 (0.80, 2.58)	0.231	
	G827C (rs2234671)	G/C	0.061	0.046	0.048	0.046	0.73 (0.41, 1.31)	0.292	0.78 (0.33, 1.83)	0.569	0.75 (0.45, 1.23)	0.253	
	C1003T (rs16858808)	C/T	0.028	0.029	0.057	0.037	1.06 (0.51, 2.23)	0.870	2.10 (0.92, 4.83)	0.080	1.36 (0.75, 2.47)	0.318	
													
													
	ZA11069G	G/A	0.053	0.036	0.008	0.028	0.57 (0.35, 1.28)	0.224	0.14 (0.02, 1.05)	0.056	0.51 (0.28, 0.96)	0.036	
													
CXCR2	ZC9316T	C/T	0.034	0.047	0.028	0.042	1.40 (0.74, 2.65)	0.303	0.80 (0.24, 2.64)	0.716	1.23 (0.68, 2.21)	0.496	
	C768T (rs11574750)	C/T	0.043	0.033	0.071	0.044	0.74 (0.37, 1.47)	0.390	1.70 (0.82, 3.52)	0.155	1.01 (0.59, 1.72)	0.969	
	T997C	T/C	0.000	0.000	0.000	0.000							
													
	ZG12229A	G/A	0.413	0.469	0.434	0.459	1.26 (0.97, 1.63)	0.078	1.09 (0.75, 1.59)	0.639	1.21 (0.97, 1.51)	0.098	
	ZT13639C	C/T	0.486	0.458	0.468	0.461	0.89 (0.70, 1.15)	0.372	0.93 (0.65, 1.34)	0.703	0.90 (0.73, 1.12)	0.363	
TLR Genes	TLR1_G1805T (rs5743618)	G/T	0.304	0.278	0.308	0.286	0.88 (0.66, 1.18)	0.404	1.02 (0.66, 1.58)	0.931	0.92 (0.71, 1.19)	0.520	
	TLR2_G2258A (rs5743708)	G/A	0.027	0.013	0.032	0.018	0.47 (0.17, 1.34)	0.160	1.18 (0.41, 3.37)	0.758	0.68 (0.31, 1.47)	0.322	
	TLR4_A896G (rs4986790)	A/G	0.064	0.056	0.000	0.040	0.88 (0.51, 1.50)	0.633			0.61 (0.36, 1.04)	0.071	
	TLR4_C1196T (rs4986791)
TLR5_C1174T(rs5744168)	C/T
C/T	0.063
0.043	0.052
0.071	0.008
0.081	0.039
0.074	0.82 (0.47, 1.43)
1.70 (1.02, 2.84)	0.486
0.041	0.12 (0.02, 0.87)
1.94 (0.97, 3.91)	0.036
0.063	0.61 (0.36, 1.04)
1.77 (1.13, 2.78)	0.071
0.012	
	TIRAP_C539T(rs8177374)
TIRAP_C558T(rs7932766)	C/T
C/T	0.144
0.232	0.163
0.217	0.186
0.202	0.170
0.213	1.16 (0.83, 1.63)
0.92 (0.68, 1.24)	0.389
0.570	1.35 (0.84, 2.19)
0.83 (0.53, 1.32)	0.214
0.438	1.22 (0.91, 1.63)
0.89 (0.68, 1.16)	0.195
0.400	
a For coding region SNPs, the name includes nucleotide numbering based on mRNA with start codon at 1.  For non-coding region SNPs, the name is from the IIPGA database (http://innateimmunity.net/IIPGA2/index_html) and designated with a 'z” prefix.  rs numbers from the dbSNP database are included when available.  A log-additive model was used for analysis.  P values ≤ 0.05 in bold.  b no ASB: <103 CFU/ml, medium ASB: >103 and <105 CFU/ml; high ASB: >105 CFU/ml.  cPolymorphism CXCR2_T997C had no variation and could not be analyzed further.
