# Supplementary material for: Genetic Variation of the Human Urinary Tract Innate Immune Response and Asymptomatic Bacteriuria in Women
Source: PLoS One. 2009 Dec 15;4(12):e8300. doi: 10.1371/journal.pone.0008300 (PMC2788705; doi:10.1371/journal.pone.0008300)
Supplement: Table S4 — CXCR1 and CXCR2 polymorphisms & clinical history of rUTI or pyelonephritis. (0.11 MB RTF) [file pone.0008300.s004.rtf]

Table S4:  CXCR1 and CXCR2 Polymorphisms & Clinical History of rUTI or Pyelonephritis

Gene	SNP	Alleles	Minor Allele Frequency	rUTI vs. Control	Pyelo vs Control	Combine vs Control	
			Control 	RUTI	Pyelo	Combined	OR, 95% CI	P a	OR, 95% CI	P	OR, 95% CI	P	
			(n=430)	(n=431)	(n=400)	(n=831)							
CXCR1	C1003T 	C/T	0.022	0.032	0.029	0.030	1.44 (0.79, 2.60)	0.234	1.31 (0.71, 2.42)	0.394	1.37 (0.80, 2.34)	0.245	
	G827C 	G/C	0.075	0.061	0.083	0.071	0.79 (0.54, 1.16)	0.229	1.11 (0.77, 1.59)	0.577	0.94 (0.69, 1.29)	0.710	
	T92G 	T/G	0.022	0.033	0.036	0.034	1.50 (0.90, 2.72)	0.175	1.62 (0.90, 2.92)	0.112	1.56 (0.92, 2.64)	0.100	
	ZA11069G	G/A	0.044	0.029	0.039	0.034	0.65 (0.54, 1.09)	0.103	0.88 (0.54, 1.42)	0.588	0.76 (0.50, 1.15)	0.199	
	rs3138060 	C/G	0.058	0.053	0.070	0.061	0.90 (0.59, 1.38)	0.641	1.22 (0.82, 1.82)	0.334	1.05 (0.74, 1.51)	0.767	
CXCR2	C768T  	C/T	0.040	0.038	0.044	0.041	0.95  (0.58, 1.55)	0.832	1.12  (0.69, 1.82) 	0.644	1.03  (0.68, 1.57) 	0.888	
	T997C b 	T/C	0	0	0	0	0	0	0	0	0	0	
	ZC9316T	C/T	0.033	0.028	0.029	0.029	0.83  (0.47, 1.47)	0.518	0.93  (0.53, 1.65) 	0.804	0.88 (0.54, 1.43)  	0.598	
	ZG12229A	G/A	0.408	0.394	0.410	0.402	0.95  (0.78, 1.15)	0.574	1.01  (0.83, 1.23) 	0.927	0.98 (0.82, 1.16)	0.778	
	ZT13639C 	C/T	0.473	0.509	0.459	0.485	1.16  (0.97, 1.40) 	0.134	0.94 (0.78, 1.14) 	0.553	1.05 (0.89, 1.24)	0.577	
a P values represent comparison of women with different clinical histories and analyzed with a log-additive model.  bPolymorphism CXCR2_T997C had no variation and could not be analyzed further.
