# Supplementary material for: Genetic Variation of the Human Urinary Tract Innate Immune Response and Asymptomatic Bacteriuria in Women
Source: PLoS One. 2009 Dec 15;4(12):e8300. doi: 10.1371/journal.pone.0008300 (PMC2788705; doi:10.1371/journal.pone.0008300)
Supplement: Table S5 — CXCR1 and CXCR2 polymorphisms & clinical history of rUTI or pyelonephritis for Caucasians only. (0.12 MB RTF) [file pone.0008300.s005.rtf]

Table S5:  CXCR1 and CXCR2 Polymorphisms & Clinical History of rUTI or Pyelonephritis for Caucasians Only

Gene	SNP	Alleles	HWE a	Minor Allele Frequency	rUTI vs. Control	Pyelo vs Control	Combine vs Control	
				Control 	RUTI	Pyelo	Combined	OR, 95% CI	P b	OR, 95% CI	P	OR, 95% CI	P	
				(n=317)	(n=339)	(n=321)	(n=660)							
CXCR1	C1003T 	C/T	0.178	0.025	0.031	0.031	0.031	1.24 (0.64, 2.40)	0.526	1.24 (0.64, 2.41)	0.532	1.24 (0.70, 2.22)	0.475	
	G827C 	G/C	1.000	0.067	0.050	0.055	0.053	0.74 (0.47, 1.18)	0.208	0.81 (0.51, 1.28)	0.365	0.77 (0.52, 1.25)	0.203	
	T92G 	T/G	0.154	0.024	0.032	0.035	0.033	1.35 (0.69, 2.64)	0.384	1.48 (0.76, 2.89)	0.246	1.41 (0.78, 2.57)	0.255	
	ZA11069G	G/A	0.611	0.058	0.033	0.047	0.040	0.55 (0.32, 0.94)	0.028	0.79 (0.48, 1.30)	0.359	0.67 (0.43, 1.03)	0.065	
	rs3138060 	C/G	0.142	0.067	0.046	0.052	0.049	0.67 (0.41, 1.09)	0.103	0.76 (0.47, 1.23)	0.26	0.71 (0.47, 1.07)	0.103	
CXCR2	C768T  	C/T	1.000	0.039	0.039	0.046	0.042	0.98 (0.56, 1.72)	0.945	1.16 (0.67, 2.01)	0.587	1.07 (0.66, 1.73)	0.788	
	T997Cc  	T/C	.	0	0	0	0	0	0	0	0	0	0	
	ZC9316T	C/T	1.000	0.042	0.031	0.036	0.033	0.73 (0.39, 1.34)	0.309	0.85 (0.47, 1.54)	0.583	0.79 (0.47, 1.32)	0.358	
	ZG12229A	G/A	0.060	0.427	0.413	0.436	0.424	0.95 (0.76, 1.18)	0.62	1.04 (0.83, 1.30)	0.769	0.99 (0.81, 1.20)	0.903	
	ZT13639C 	C/T	0.315	0.470	0.503	0.470	0.487	1.14 (0.92, 1.42)	0.232	1.00 (0.80, 1.25)	0.989	1.07 (0.89, 1.30)	0.477	
 a HWE=Hardy Weinberg Equilibrium P value, a value>0.001 indicates that polymorphism is in Hardy-Weinberg Equilibrium.
b P values represent comparison of genotype frequencies analyzed with a log-additive model among women with different clinical UTI histories
cPolymorphism CXCR2_T997C had no variation and could not be analyzed further.
